# A micro-CT-based standard brain atlas of the bumblebee

**DOI:** 10.1007/s00441-021-03482-z

**Published:** 2021-06-28

**Authors:** Lisa Rother, Nadine Kraft, Dylan B. Smith, Basil el Jundi, Richard J. Gill, Keram Pfeiffer

**Affiliations:** 1 Department of Behavioral Physiology and Sociobiology, Biocenter, University of Würzburg, 97074 Würzburg, Germany; 2 Department of Life Sciences, Imperial College London, Silwood Park, Buckhurst Road, Ascot, Berkshire, SL5 7PY UK

**Keywords:** *Bombus terrestris*, Insect standard brain atlas, Iterative shape averaging, Neuropils, Reconstruction

## Abstract

In recent years, bumblebees have become a prominent insect model organism for a variety of biological disciplines, particularly to investigate learning behaviors as well as visual performance. Understanding these behaviors and their underlying neurobiological principles requires a clear understanding of brain anatomy. Furthermore, to be able to compare neuronal branching patterns across individuals, a common framework is required, which has led to the development of 3D standard brain atlases in most of the neurobiological insect model species. Yet, no bumblebee 3D standard brain atlas has been generated. Here we present a brain atlas for the buff-tailed bumblebee *Bombus terrestris* using micro-computed tomography (micro-CT) scans as a source for the raw data sets, rather than traditional confocal microscopy, to produce the first ever micro-CT-based insect brain atlas. We illustrate the advantages of the micro-CT technique, namely, identical native resolution in the three cardinal planes and 3D structure being better preserved. Our *Bombus terrestris* brain atlas consists of 30 neuropils reconstructed from ten individual worker bees, with micro-CT allowing us to segment neuropils completely intact, including the lamina, which is a tissue structure often damaged when dissecting for immunolabeling. Our brain atlas can serve as a platform to facilitate future neuroscience studies in bumblebees and illustrates the advantages of micro-CT for specific applications in insect neuroanatomy.

**Supplementary information:**

The online version contains supplementary material available at 10.1007/s00441-021-03482-z.

## Introduction

Standard brain atlases have become an important tool in insect neuroanatomy, serving as an important reference for neurobiological studies. Having a clear understanding of the relative positioning, shape, and structure of the brain reveals insights into its form and function, as well as providing the framework for mapping neuronal networks and understanding neuron branching patterns (e.g., Brandt et al. 2005; Heinze and Homberg 2008; Kurylas et al. 2008; Chiang et al. 2011; Peng et al. 2011; Heinze et al. 2013). The generation of standard brain atlases is thus important to allow comparative anatomical studies, with standard brain atlases currently available for a variety of model insect species: flies (*Drosophila melanogaster*: Rein et al. 2002; Peng et al. 2011; Ostrovsky and Jefferis 2014; Arganda-Carreras et al. 2018; Bogovic et al. 2020), beetles (*Tribolium castaneum*: Dreyer et al. 2010), locusts (*Schistocerca gregaria*: Kurylas et al. 2008), wasps (*Nasonia vitripennis*: Groothuis et al. 2019), and lepidopteran species (*Manduca sexta*: el Jundi et al. 2009; *Heliothis virescens*: Kvello et al. 2009; *Agrotis infusa*: Adden et al. 2020).

Despite bees being speciose, and an ecologically as well as economically important insect pollinator group, only the honeybee *Apis mellifera* has a standard brain atlas (Brandt et al. 2005). Honeybees are a domesticated and highly managed species complex, exhibiting aspects of physiology and behavior that may not be representative of other wild bees (Fischer et al. 2016; Brunet et al. 2019). Creating a standard atlas for other bee groups, such as the bumblebees which are being increasingly considered as a model organism in insect neurobiology research (e.g., Paulk and Gronenberg 2008; Paulk et al. 2008; Pfeiffer and Kinoshita 2012; Stone et al. 2017), can therefore facilitate bee and hymenoptera comparative neurobiology.

Social bumblebees like *Bombus terrestris* can be relatively easily maintained in the lab making them an amenable model to study insect cognitive behavior (e.g., Sherry and Strang 2015; Loukola et al. 2017), olfactory and visual systems (e.g., Paulk et al. 2009; Riveros and Gronenberg 2009; Sommerlandt et al. 2014; Smith et al. 2020), and spatial orientation (e.g., Jacobs-Jessen 1959; Wellington 1974; Dyhr and Higgins 2010; Sovrano et al. 2012; Crall et al. 2015; Ravi et al. 2019). These behaviors determine key components of insect life history, such as mate choice, interaction with conspecifics, predator avoidance, and foraging, and so are of great interest to biologists from a range of disciplines. Therefore, a common reference frame for studying the neuronal underpinnings of these behaviors, a standard brain atlas, is critically needed.

All insect standard brain atlases created so far, however, have been based on a combination of immunocytochemical neuropil labeling followed by confocal microscopy. Although this is a well-established and reliable method, there are some disadvantages. For instance, the necessity to dissect the brain out of the head capsule for staining can lead to a misalignment or distortion of the tissue. This method is thought to disproportionately affect, e.g., the optic and antennal lobes due to their fragility (Groothuis et al. 2019). Another drawback pertains to confocal microscopy. The resulting data stack typically has a non-isotropic resolution, which is due to physical limitations that lead to a much higher lateral than axial resolution. For 3D reconstruction of neurons, it is desirable to have isotropic voxels, which is usually achieved by down-sampling of the data, leading to a loss of lateral resolution (Kurylas et al. 2008; Jenett et al. 2012; Heinze et al. 2013; Aso et al. 2014; el Jundi et al. 2018; el Jundi and Heinze 2020). A technology that can eliminate both problems, however, is micro-computed tomography (micro-CT). Employing this technology makes it possible to generate images that are isotropically resolved. Furthermore, micro-CT does not require dissection of the brain from the head capsule leaving the brain embedded in, and protected by, the surrounding tissue maintaining its natural shape and stereo geometry.

The use of micro-CT imaging is an emerging technique in research, which has provided new insights into the form and function of microscopic structures that would otherwise be inaccessible or damaged through dissection. This has made micro-CT scans particularly beneficial for research on small invertebrates (bees: Ribi et al. 2008; Taylor et al. 2016, flies: Mokso et al. 2013; Sombke et al. 2015, ants: Garcia et al. 2017, and other arthropods: Handschuh et al. 2013; Sombke et al. 2015; Shearer et al. 2016; Baird and Taylor 2017; Castejón et al. 2018). While micro-CT has some advantages over light-microscopy techniques, high-resolution visualization of neuronal morphology is to date only possible with confocal laser scanning microscopy or light-sheet microscopy. Recent work by Smith et al. (2016) optimized a protocol for micro-CT scanning of the bumblebee brain, and further illustrated its use to understand how changes to brain structure can affect behavior (Smith et al. 2020). Taking advantage of these recent developments, we here construct the first ever insect standard brain atlas based on micro-CT images from the bumblebee study of Smith et al. (2020). We present an atlas assembled from 3D reconstructions of 30 neuropil areas of ten adult *B. terrestris* worker brains using the iterative shape averaging method (Kurylas et al. 2008; Rohlfing and Maurer 2003). Our study contributes a framework for future neuroanatomical work in the bumblebee. We also demonstrate the advantages and limitations of the micro-CT method, by comparing micro-CT images to data obtained through anti-synapsin labeling and confocal microscopy.

## Material and methods

### Bumblebee husbandry

Bumblebee (*Bombus terrestris*) colonies were obtained from two commercial suppliers (Biobest NV, Belgium and Koppert B.V., Berkel en Rodenrijs, NL). Worker bumblebees sampled for micro-CT imaging of the brain were from Biobest colonies delivered to Silwood Park, Imperial College London (UK) which were kept in a controlled environment room at 23 °C and 60% relative humidity (rH) under continual red light and provisioned ad-libitum food (for further details, see Smith et al. 2020). Workers sampled for the anti-synapsin staining and confocal microscopy of the brain were from Koppert colonies delivered to the University of Würzburg (Germany) and kept in large climate chambers at 25 °C and 55% rH under white light (12:12 LD) and provisioned ad-libitum food.

### Micro-CT imaging

The following protocols and resultant micro-CT scans are taken from a previous study by the authors (Smith et al. 2020). From three control colonies, 10 adult workers were removed (2–4 per colony), placed on ice, and sacrificed by decapitation of the head using a disposable surgery scalpel. Each head was then immediately placed inside separate 1.5-ml tubes containing 70/30% ethanol/water solution and stored at 5 °C, and the thorax width (intertegula width) of the remaining bodies was measured (± 0.01 mm). Of the 10 workers, five were aged 3 days post pupal emergence, and five were 12 days, with these specific individuals showing a similar body size and volume of neuropils between the age cohorts (see Supplementary Table S1; Supplementary Table S2; and Supplementary Fig. S3).

To stain in situ the brain prior to micro-CT scanning, the head was removed from its storage tube, submerged in a 0.5% phosphotungstic acid (PTA) solution (0.5 mg/ml conc. in 70% ethanol), and left for 7 days. On the eighth day, we removed the heads and placed them inside a plastic straw for scanning (two heads per scan; see Smith et al. 2016). Scanning was performed using a Nikon Metrology HMX ST 225 system (Nikon Metrology, Tring, UK), with cone beam projection system and four megapixel detector panel (max. output of 225 kV for the reflection target and max. current output of 2000 μA). The focal spot size was 5 μm, and the exposure ranged from 0.25 to 5.6 frames s^−1^. Raw micro-CT data for each brain was reconstructed using CTPro 2.1 software (Nikon Metrology, Tring, UK) and processed using VG Studio Max 2.1 (Volume Graphics GmbH, Heidelberg, Germany). For the 3D reconstructions, we re-oriented each brain to the same optimum plane-of-view and re-sliced into a new series of 2D images. Scan resolution varied between 3.5 and 3.9 μm, and we exported as 8-bit BMP image series with slices for each of the ten brains standardized to a voxel size representing the upper limit of this range (3.9 × 3.9 × 3.9 μm^3^).

### Three-dimensional average shape atlas

**Fig. 1:**
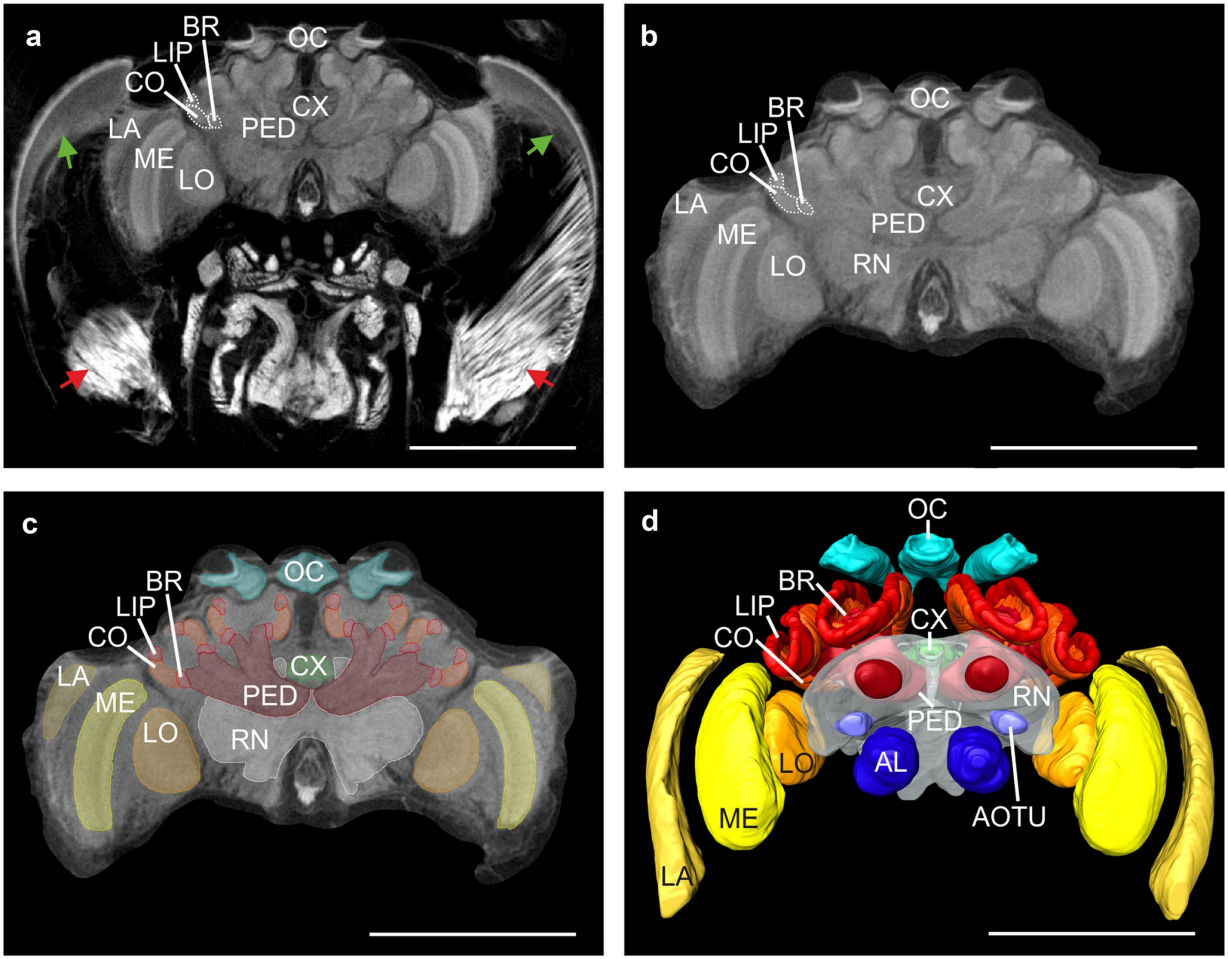
Workflow for reconstruction of neuropils from micro-CT data of *Bombus terrestris*. (**a**) Virtual frontal 2D slice from the micro-CT data showing the position of the brain in the head capsule. Red arrows: muscles; green arrows: retina. (**b**) Same slice as in (**a**) but cropped to exclude all non-neuronal tissues. (**c**) The cropped slice with all neuropil regions of interest manually labeled. The color code of the neuropils is consistent with Brandt et al. (2005) and Kurylas et al. (2008). (**d**) Surface model of an individual brain reconstruction with all neuropils labeled. The remaining neuropils (RN) were labeled semi-transparent to make the central complex (CX) better visible. Neuropils: antennal lobes (AL), anterior optic tubercle (AOTU), basal ring (BR) central complex (CX), collar (CO), lamina (LA), lip (LIP), lobula (LO), medulla (ME), ocellar synaptic plexi (OC), peduncle (PED), and remaining neuropils (RN). Scale bars = 1000 µm

For neuropil segmentation, the 3D software Amira (Thermo-Fisher, Version 6.2) was used. To get the best possible result from the averaging procedure, we first extracted the voxels containing brain tissue data from the micro-CT scans of the entire head capsule. This was done by reconstructing the 3D-shape of the brain surface and excluding all voxels outside of it. To this end, the surface area of the brain was marked at 10 to 15 slices of the brain tissue in each of the three planes (XY, XZ, YZ), creating a scaffold that delimited the desired area. Then the “Wrap” function was used to mark the complete area in the grid and to delineate the border of the entire brain. Next, the wrapped area was cropped using the “Arithmetic” function, by changing the grey value of all voxels outside the brain area to 0 (black). The dynamic range of the cropped neuronal tissue was usually just a subset of the 256 gray values available in the raw images, especially due to the bright appearance of muscle tissue (Fig. 1a). For best registration results, it is desirable that both reference and floating image cover the same dynamic range. Therefore, data sets were normalized to cover a range of grey values from 0 to 255, following the cropping procedure. Next, we segmented the neuropils in all brains by using the “Segmentation” editor of Amira. The borders of the individual neuropils were manually marked in the XY plane approximately every 4–6 slices. We included all neuropils that were recognizable in the micro-CT scans. These were laminae (LA), medullae (ME), lobulae (LO), antennal lobes (AL), ocellar synaptic plexi (OC), protocerebral bridge (PB), upper and lower division of the central body (CBU, CBL), noduli (NO), anterior optic tubercles (AOTU), collar of the lateral and medial mushroom body calyces (LCO, MCO), basal ring of the lateral and medial mushroom body calyces (LBR, MBR), lip of the lateral and medial mushroom body calyces (LLIP, MLIP), pedunculus including the medial and vertical lobes (PED) of the mushroom bodies, and the remaining neuropils (RN). Segmentations were completed with the “Interpolation” function and manually checked for accuracy. Neuropil volumes were measured using Amira’s “MaterialStatistics” function.

### Standardization

For standardization, we reconstructed ten *B. terrestris* brains based on the micro-CT images. To obtain an average standard brain, the standardization was carried out by applying the iterative shape averaging procedure (ISA) to the micro-CT data of the brain tissue using the computational morphometry toolkit (CMTK, Version 3.3.1 (http://nitrc.org/projects/cmtk); Kurylas et al. 2008; Rohlfing and Maurer 2003). All calculations were carried out on the high-performance Linux cluster Julia at the University of Würzburg, Germany. The ISA protocol is a two-step procedure, in which an affine registration is followed by repeated elastic registrations (Supplementary Fig. S1). Both registration processes used normalized mutual information as the metric. First, a reference brain was chosen, which was the most representative in terms of volume (close to the median volume). Then, the nine remaining brains were aligned onto the template using two affine registration processes, first with six degrees of freedom (translation, rotation) followed by nine degrees of freedom (translation, rotation, scaling). This procedure compensated for differences in size, position, and rotation between the individual brains. After affine registration, all ten data sets were averaged, and the resulting coarse average brain served then as the template for the subsequent elastic registrations. Elastic registration applies local transformations to each brain, thereby optimizing the similarity between images. In this process, a three-dimensional grid is applied to the image stack and this grid is warped locally using a B-spline free form deformation model to match the local features of the current brain to the template brain. After elastic registration of all ten brains, a new average was generated which served as the new template for the next iteration. This process was done five times in total, with finer grids in each round. To obtain a standardized average of the segmented brains (label fields), the registration parameters for each reconstructed brain were applied to the corresponding label fields, deforming them in the same way as the original micro-CT data. The result of this operation was ten label fields with very similar shape. In a final step, a shape-based average was computed from these ten segmentation data sets using signed Euclidean distance maps to obtain the final standard atlas of neuropils (Rohlfing and Maurer 2007).

The registration processes use the mutual information content of the image. This usually leads to a better registration performance of neuropils that are large and/or have high contrast outlines, like the mushroom bodies or the optic lobe neuropils. Small neuropils in the central brain, like the central complex, are represented by fewer voxels and have therefore less information content and consequently less weight in the registration process. We therefore standardized these neuropils separately with three iterations of the elastic registration process. Next, we used an affine registration with six degrees of freedom (translation and rotation) to register the central complex neuropils into the final brain model.

### Neuron registration

To visualize central complex input neurons in the bumblebee standard brain, we iontophoretically injected Neurobiotin (Vector Laboratories, Burlingame, GB). The brain was then dissected and fixated overnight in a sodium phosphate buffer containing 4% paraformaldehyde, 0.2% saturated picric acid, and 0.25% glutaraldehyde. The brain was subsequently washed in 0.01 M phosphate-buffered saline (PBS) for 4 × 15 min and incubated with Alexa568 conjugated to streptavidin (Molecular Probes, 1:1000 in PBS with 0.5% Triton X-100 detergent (PBT)) for 3 days at 4 °C. Afterwards, the brain was rinsed (2 × 15 min in PBT and 3 × 15 min in PBS) and dehydrated in an ascending ethanol series. Dehydration, clearing, and mounting of the brain accurately followed the steps described for the synapsin immunostaining (see below). The brain was scanned with a confocal laser scanning microscope (Leica TCS SP8, Leica Microsystems, Wetzlar, Germany) using a HeNe-laser and a 20× water-immersion objective (HC PL APO CS2 20x/0.75 IMM).

To register the injected neurons into the bumblebee standard atlas, we first reconstructed the lower division of the central body and the ipsilateral antennal lobe and pedunculus based on background fluorescence staining. These 3D reconstructed neuropils were then affinely registered into the corresponding 3D neuropils of the standard atlas with 12 degrees of freedom (translation, rotation, scaling, shearing) in the software Amira. Next, the standard brain data was resampled to match the resolution of the confocal scans. While this does not affect the registration accuracy, which is limited to the original resolution of the standard brain, it retains the higher spatial resolution of the confocal image. This was followed by an elastic registration of the neuropils onto the standard neuropils using the warp tool in CMTK. The resulting registration parameters were then applied to the confocal data of the injected neuron using the reformatx tool in CMTK.

### Bumblebee synapsin immunostaining

Bumblebee worker brains were dissected in ice-cold HEPES-buffered saline (HBS; 150 mM NaCl, 5 mM KCl, 5 mM Ca_2_Cl, 25 mM Sucrose, 10 mM HEPES; pH 7.2) and immediately fixed in ice-cold ZnFA (1% Paraformaldehyde, 18.4 mM ZnCl_2_, 135 mM NaCl, 35 mM Sucrose) for 20 h at room temperature. Afterwards, they were washed in HBS (8 × 30 min) and Tris-HCl (3 × 10 min) and permeabilized in a solution of DMSO and methanol (20:80; 85–90 min) followed by another washing step in Tris-HCl (3 × 10 min). To block unspecific binding sites, the brains were incubated in 5% Normal Goat Serum (NGS; DIANOVA GmbH, Hamburg, Germany) in 0.01 M phosphate-buffered saline (PBS; 137 mM NaCl, 2.7 mM KCl, 8 mM Na_2_HPO_4_, 1.4 mM KH_2_PO_4_; pH 7.2) with 0.3% Triton-X 100 (PBT) and 1% DMSO at 4 °C overnight. For primary antibody labeling, we used a monoclonal antibody against the *Drosophila* synaptic‐vesicle‐associated protein synapsin I (3c11; kindly provided by E. Buchner, University of Würzburg, Germany). Brains were incubated in 3c11 (1:50) in PBT with 1% NGS for 8 days at 4 °C. After washing the brains in PBT (8 × 30 min), a CF633-conjugated goat anti-mouse secondary antibody (20121, Biotium Inc., Fremont, CA, USA) was applied (1:250 in PBT and 1% NGS) for another 8 days at 4 °C. After rinsing in PBT (6 × 30 min) and 0.01 M PBS (2 × 30 min), the brains were dehydrated in an ascending ethanol series (50%, 70%, 90%, 95%, 2 × 100%; 15 min each). For clearing, the brains were first transferred into a 1:1 methyl salicylate/100% ethanol solution (15 min) and then moved into pure methyl salicylate for at least 60 min. Custom chambers for permount embedding were built by stacking 13 spacers (Reinforcement Rings, Avery Zweckform GmbH, Oberlaindern, Germany) on a coverslip. The brains were then mounted in a drop of permount and sealed with another coverslip. The samples were scanned with a confocal laser scanning microscope (Leica TCS SP8, Leica Microsystems, Wetzlar, Germany) using a 638-nm Diode Laser and a 20× water-immersion objective (HC PL APO CS2 20×/0.75 IMM) with a digital zoom of 0.75.

### Nomenclature

Naming of the neuropils follows the nomenclature suggested by Ito et al. (2014) wherever possible. The terms, dorsal, ventral, anterior, and posterior refer to the animal’s body axes rather than the neuraxis.

## Results

The original micro-CT scans showed the entire head of the bumblebees, including tissues and structures around the brain itself, like muscles, retina, fat body, glands, and tracheae (Fig. 1a). Those structures were excluded from the registration process to reduce the size of the data sets and avoid registration problems due to variability in the spatial arrangement of some of these structures (Fig. 1b). The resulting data sets were then used for the 3D reconstruction of neuropils (Fig. 1c, d). We reconstructed all areas of the bumblebee brain that we could clearly demarcate. Neuropils, which occur on both hemispheres, were separated into “left” and “right.” In total, we reconstructed 30 neuropils. These include the lamina (LA), the medulla (ME), and the lobula (LO) of the optic lobes; the mushroom bodies (peduncle (PED), the collar of the calyx (CO), the basal ring of the calyx (BR), and the lip of the calyx (LIP)); the central complex (protocerebral bridge (PB), the upper and lower division of the central body (CBU, CBL), and the paired noduli (NO)); and the anterior optic tubercle (AOTU), the ocellar synaptic plexi (OC), the antennal lobes (AL), and the remaining neuropils (RN).

**Fig. 2:**
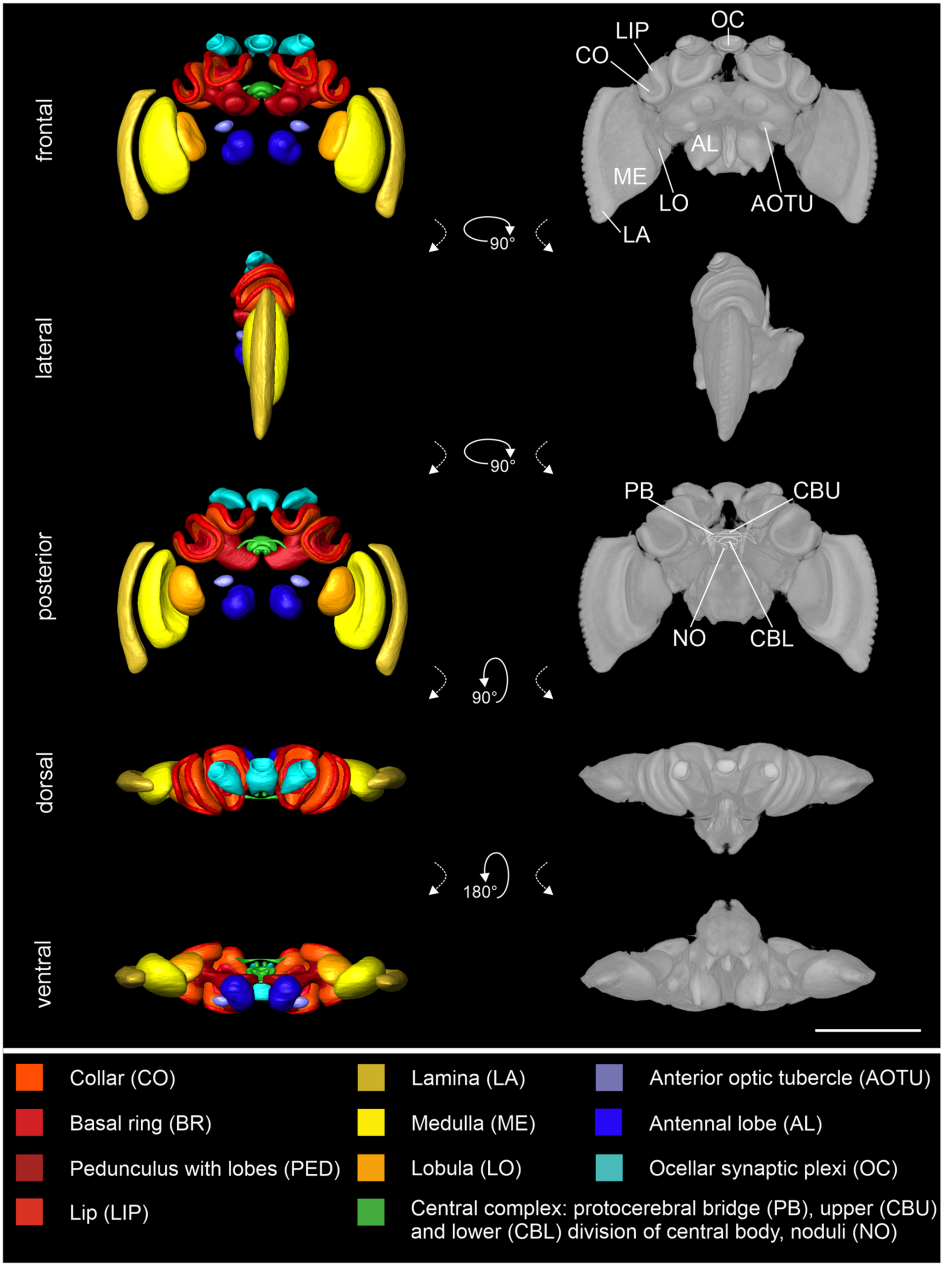
Standard brain atlas of *Bombus terrestris*. Left column: Shape-based average of surface reconstruction from a frontal, lateral, posterior, dorsal, and ventral perspective. The color code at the bottom represents the colors of the reconstructed neuropils. Right column: Direct volume rendering of averaged raw data from a frontal, lateral, posterior, dorsal, and ventral perspective. For better visibility of the neuropils in the central brain, the remaining neuropils (RN) were excluded in this figure. The whole standard including the RN is provided in the supplementary information (Supplementary Fig. S2). Scale bar = 1000 µm

The result of the iterative shape averaging (ISA) protocol is a 3D standard atlas of the reconstructed neuropils (Fig. 2, left column) as well as an average of the micro-CT data (Fig. 2, right column). For better visibility of the neuropils in the central brain, the remaining neuropils (RN) were excluded in this figure. The whole standard including the RN is provided in the supplementary information (Supplementary Fig. S2).

### Morphology

#### Optic lobes

**Fig. 3:**
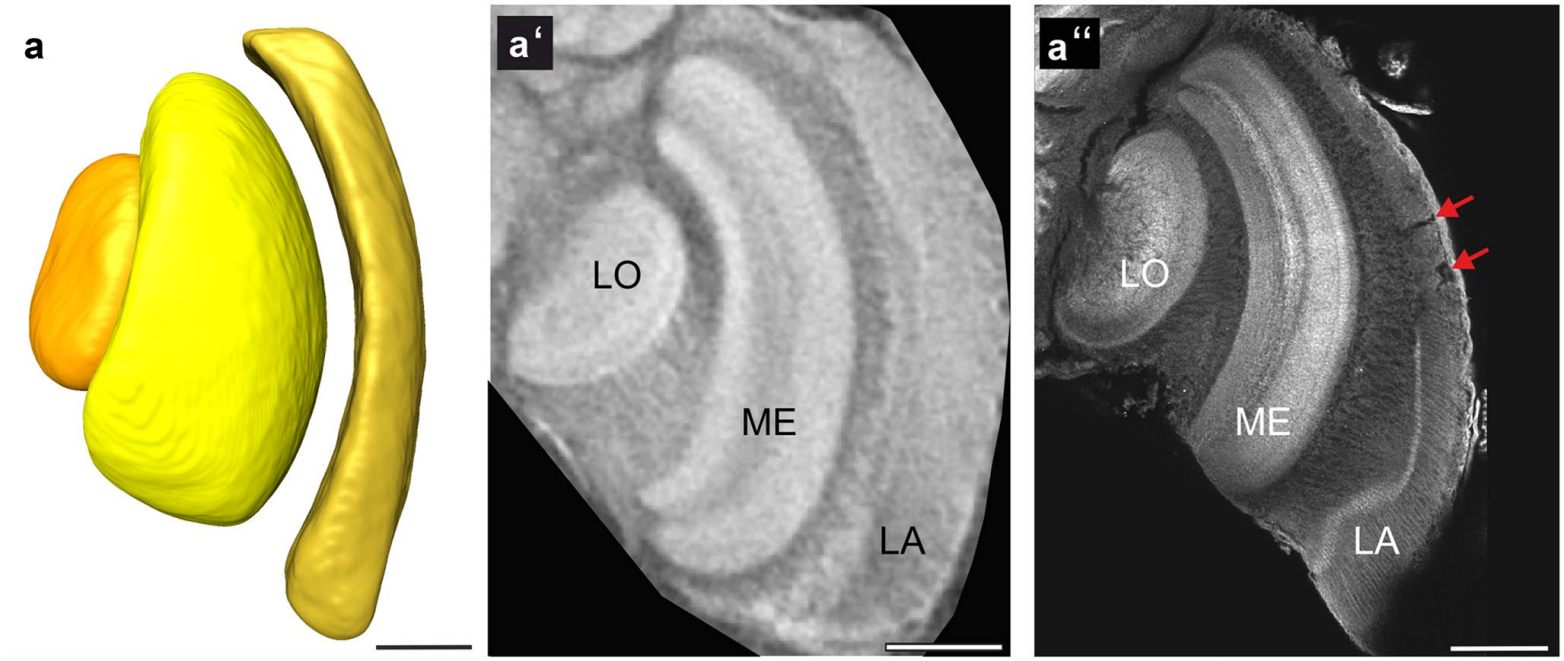
Optic Lobes. (**a**) Frontal view of a shape-based average of surface reconstruction showing the primary visual neuropils including lamina (LA), medulla (ME), and lobula (LO). (**a′**) Frontal view of one slice of the micro-CT scans showing an optic lobe. (**a′′**) Frontal optical section (confocal image) of optic lobe, stained against synapsin. Red arrows show damage of the LA that occurred during dissection. Scale bars = 200 µm

The optic lobes (OLs; Fig. 3) are divided into three neuropils: the lamina (LA), the medulla (ME), and the lobula (LO). As in other Hymenopterans, a lobula plate is missing in bumblebees. The LA is the outermost optic neuropil and is a narrow, elongated curved structure. It extends beyond the dorsal and ventral edges of the ME (Fig. 3) but is slimmer along its anterior-posterior axis (Fig. 2). Using the micro-CT technique, the lamina could be reconstructed in its natural shape in each of the ten specimens, without any strains or ruptures that often occur during the traditional staining method (Fig. 3a″, red arrows), which requires the removal of the retina for confocal scans. The second visual processing stage, the ME, is positioned between the LA and the LO. The ME is the largest neuropil of the optic lobes and is divided into an inner and outer part. Layering of the medulla was apparent in its lateral area, but the layers were less defined than in the anti-synapsin staining (Fig. 3a′, a″). The smallest of the optic lobes neuropils, the LO, is situated medial to the ME. As in the ME, layering of the LO was visible in the CT-scans, but less pronounced than in the anti-synapsin staining.

#### Ocellar synaptic plexi

**Fig. 4:**
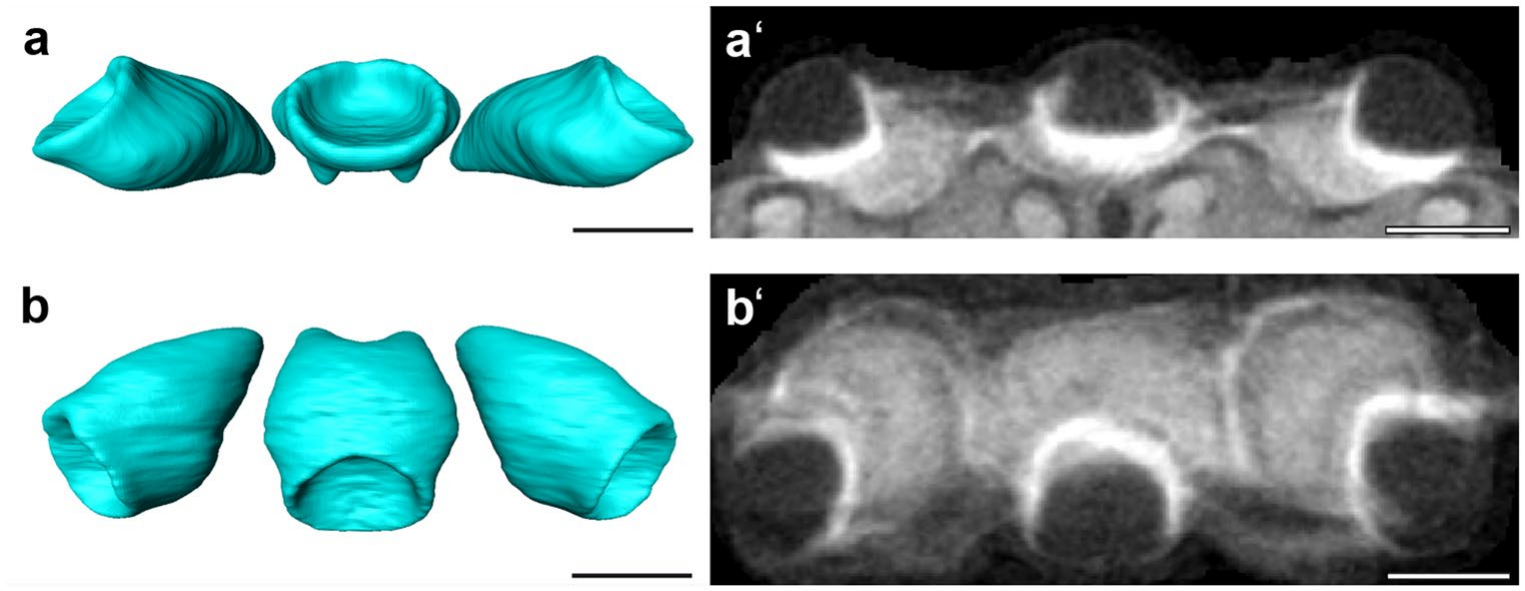
Ocellar synaptic plexi. Shape based average of surface reconstruction of the ocellar synaptic plexi (OC) of *B. terrestris* standard brain atlas in frontal view (**a**) and dorsal view (**b**). Micro-CT scans of the OC in frontal view (**a′**) and dorsal view (**b′**). Scale bars = 200 µm

The three ocellar synaptic plexi (OC; Fig. 4) of *B. terrestris* protrude dorsally from the brain surface. In *B. terrestris*, the OC are arranged next to each other almost in a straight line, with the median ocellus located about half an ocellar width more anteriorly than the lateral ones. The micro-CT data show that the longitudinal axis of the ocellar synaptic plexi is tilted anteriorly and, in the lateral ocellar plexi sideways.

#### Mushroom bodies

Each MB consists of a lateral and medial calyx, the pedunculus, a vertical, and a medial lobe. Each calyx comprises three subunits: the lip (LIP), the collar (CO), and the basal ring (BR). The calyces are connected to the pedunculus (PED), which bifurcates into the vertical lobe (VL) that extends to the anterior brain surface and the medial lobe (ML) that extends to the centerline of the brain.

**Fig. 5:**
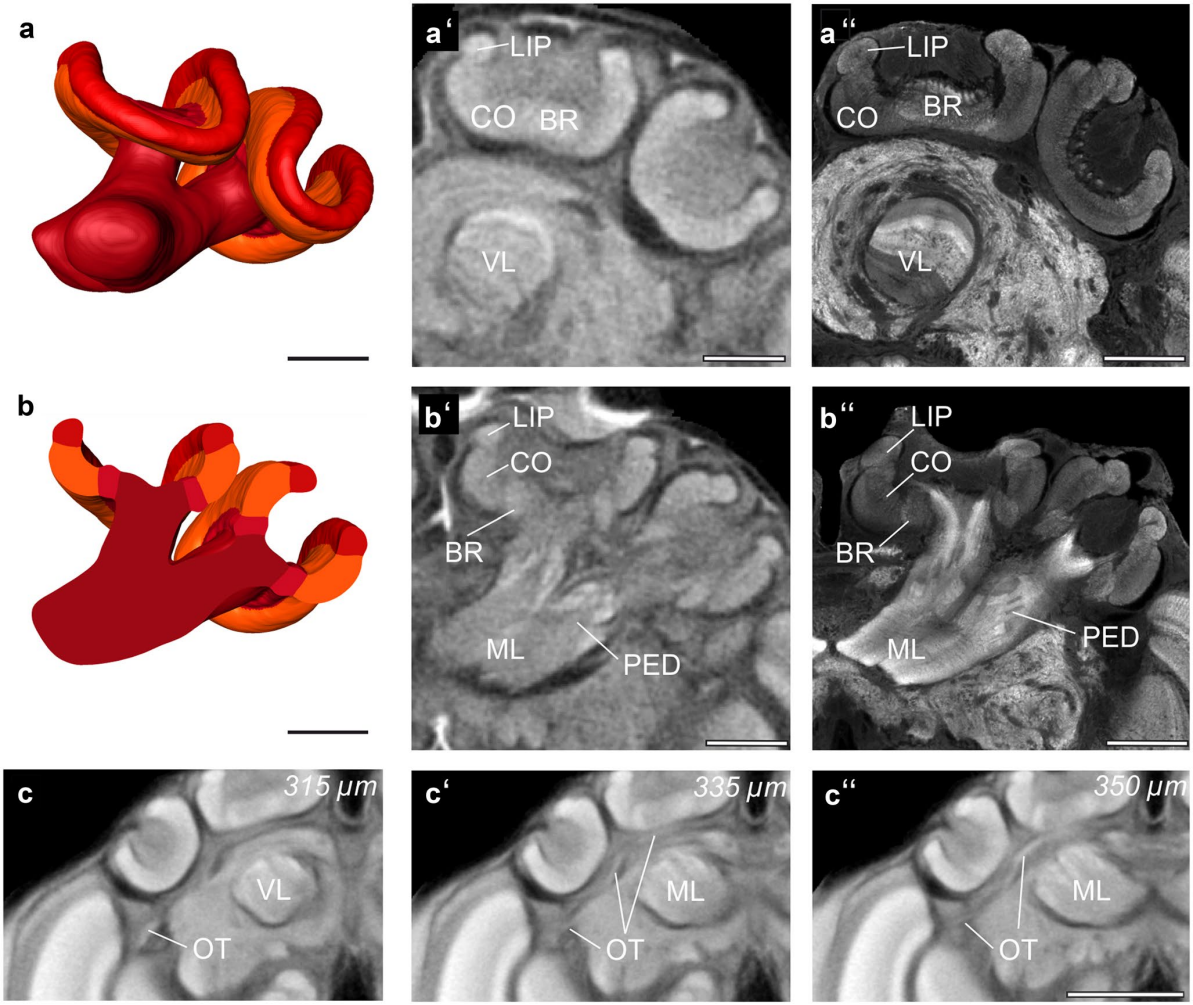
Mushroom bodies. (**a**) Shape-based average of surface reconstruction of the mushroom body (MB) with pedunculus (PED), vertical lobe (VL), medial lobe (ML), and the calyx (basal ring: BR, collar: CO, lip: LIP). (**b**) Virtual section through 3D reconstruction shown in (**a**). (**a′** and **b′**) Anterior view of micro-CT scans at two different levels showing the compartments of the MB. (**a′**) Anterior slice shows the BR, CO, LIP, and VL. (**b′**) Posterior slice shows the BR, CO, LIP, and PED. (**a″** and **b″**) Confocal image (frontal optical slice) of the MB, stained with an antiserum against synapsin. Optical section levels correspond to those in (**a′**) and (**b′**). (**c**, **c′**, and **c″**) The optic tracts (OT) consisting of the anterior superior optic tract (ASOT), the anterior inferior optic tract (AIOT), and the lobula optic tract (LOT). The tracts are shown here in different depths of the standard brain micro-CT data. Since they run in parallel, they cannot be distinguished here. Scale bars = 200 µm (**a-b″**), 500 µm (**c-c″**)

The comparison between the micro-CT data and synapsin stainings shows, that both methods reveal similar details of the mushroom body, including the layering in the VL (see Fig. 5a′, b′) compared to Fig. 5a″, b″). In the synapsin staining, it is possible to subdivide the collar into the dense and loose collar region. Beside the subunits of the MBs, the micro-CT data prominently exposes larger tracts. Tracts are bundles of axons that link different neuropils. In the area of the MBs, an optic tract (OT) that connects the OL to the MB could be identified. This large diameter (mean = 23.5 ± 1.5 µm) fiber tract consists of the anterior superior optic tract (ASOT), the anterior inferior optic tract (AIOT), and the lobula optical tract (LOT) (Ehmer and Gronenberg 2002). These three tracts run in parallel and could therefore not be discerned at the available level of resolution. All three tracts provide visual input into the CO coming either from the ME (ASOT, AIOT) or the LO (LOT) (Fig. 5c–c″).

#### Central complex

**Fig. 6:**
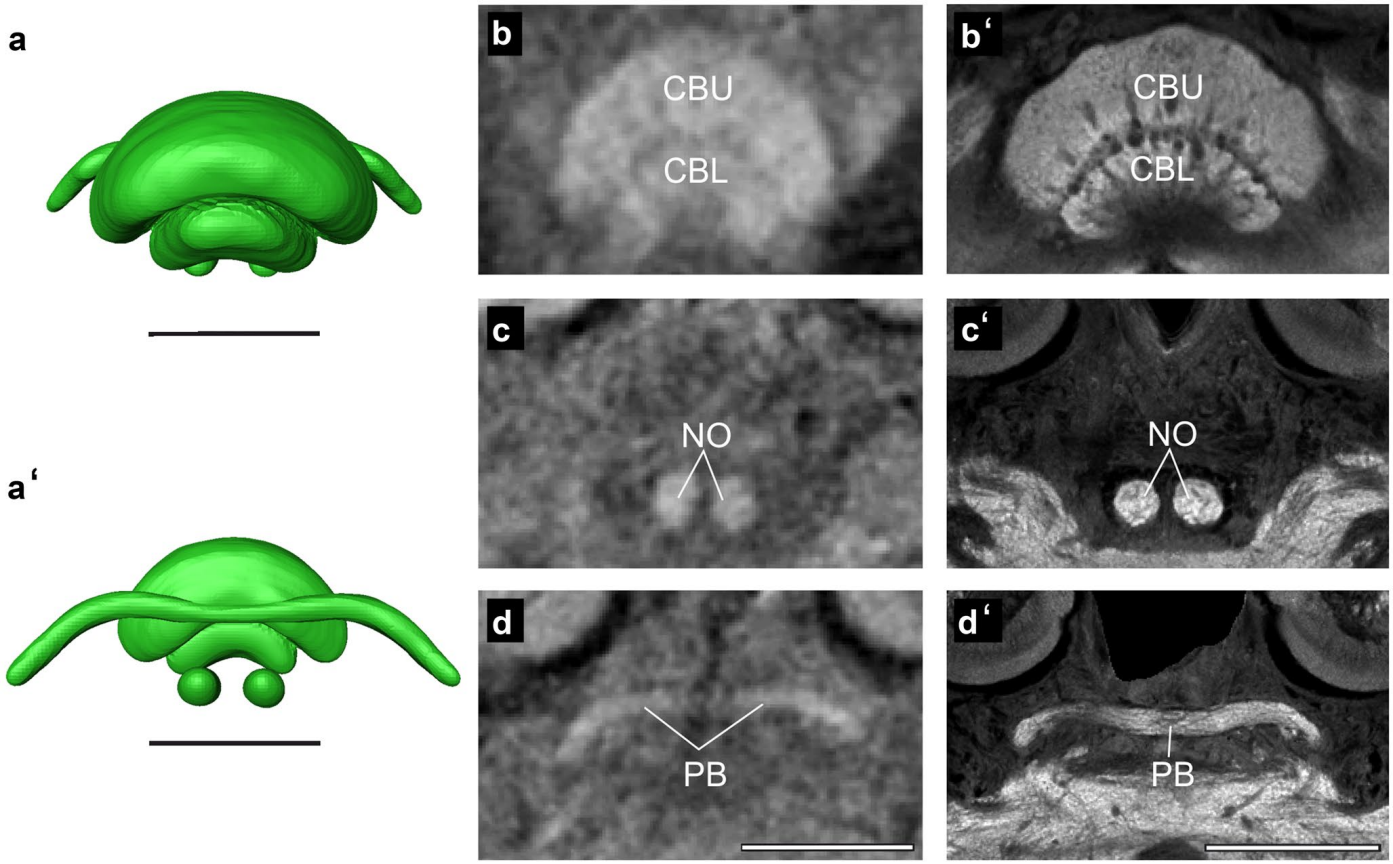
Central Complex. (**a**) Shape-based average of central complex surface reconstruction showing the upper division of the central body (CBU), the lower division of the central body (CBL), the protocerebral bridge (PB), and the paired noduli (NO). (**a′**) Posterior view of (**a**) shows the complete PB and NO. (**b**, **c**, and **d**) Frontal view of CX in different depths of micro-CT scan shows all neuropils comprising the CX. (**b′**, **c′**, and **d′**) Anti-synapsin immunolabeling showing the CX neuropils at similar depths. Scale bars = 200 µm

The central complex (CX; Fig. 6), a group of midline-spanning neuropils, is located in the center of the bumblebee brain. The CX consists of the upper division of the central body (CBU; also called fan-shaped body in some insects), the lower division of the central body (CBL; also called ellipsoid body in some insects), the protocerebral bridge (PB), and the paired noduli (NO). The CBU lies dorsally to the tip of the ML and is the largest substructure of the CX. The CBL is embedded ventrally in the arch of the CBU. It is smaller than the CBU. The PB is located posteriorly to the CBU and has a handlebar-like shape. It is the third largest neuropil in the CX. The smallest part of the CX are the NO which are located posterior and ventrally to the central body.

#### Anterior optic tubercle

**Fig. 7:**
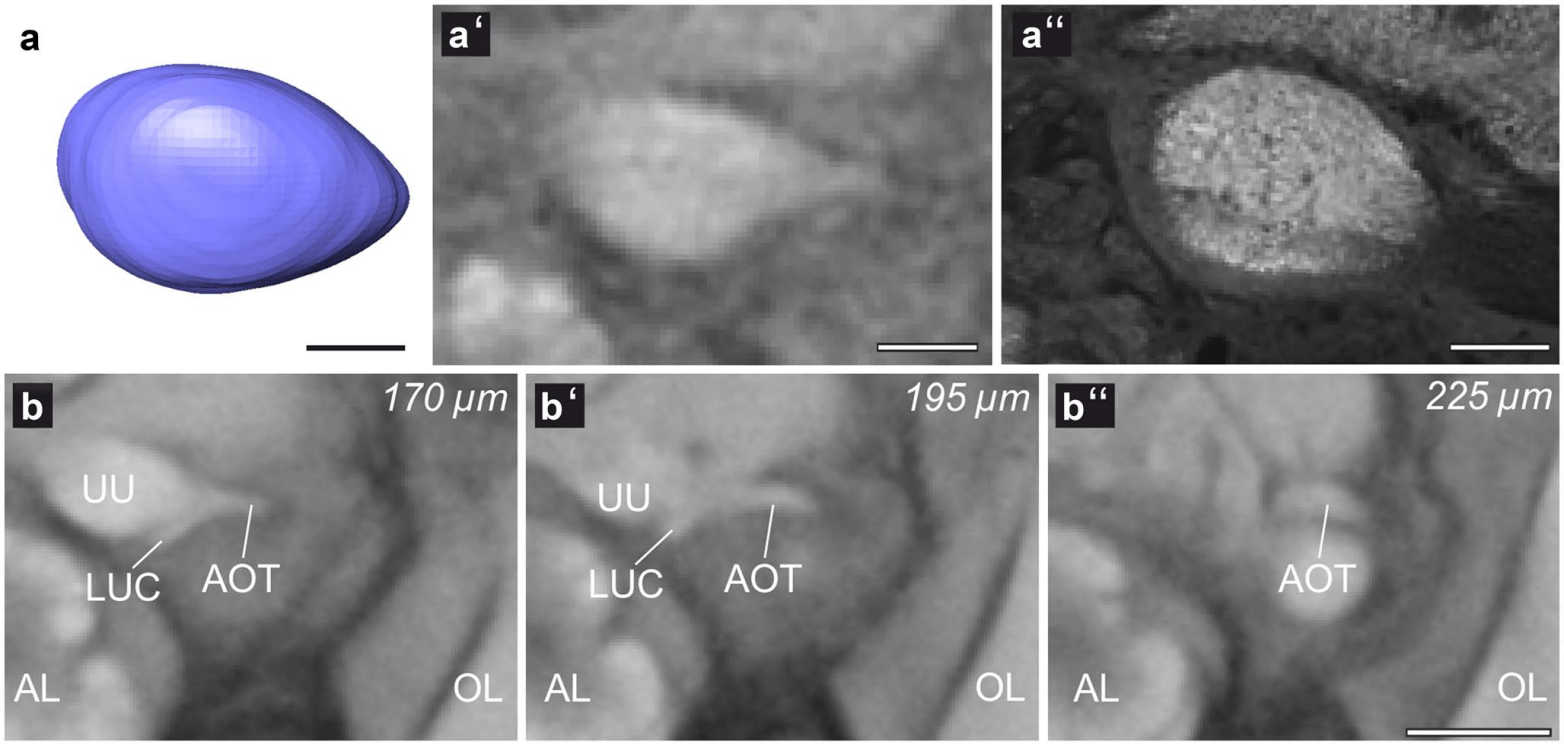
The anterior optic tubercle. (**a**) Shape-based average of surface reconstruction of the right anterior optic tubercle (AOTU). (**a′**) One slice of the micro-CT scan of the left AOTU in frontal view. (**a″**) Optical horizontal plane of a synapsin staining of the left AOTU. (**b**, **b′**, and **b″**) Frontal slices of the standardized grey data of the micro-CT scans in different planes. The figures present the course of the anterior optic tract (AOT) from the AOTU to the optic lobes (OL). AL antennal lobe, LUC lower unit complex of the AOTU, UU upper unit of the AOTU. Scale bars = 50 µm (**a–a″**), 200 µm (**b–b″**)

The anterior optic tubercles (AOTUs; Fig. 7) are located dorsally to the ALs. Each AOTU extends approximately from the midline of the AL to its lateral edge. It consists of a larger upper and a smaller lower unit. The subunits were not easy to distinguish in all ten specimens and were therefore reconstructed as one neuropil. However, in the averaged data set, the lower unit complex is clearly set apart from the upper unit of the AOTU (Fig. 7b, b′). Furthermore, the micro-CT scans clearly show the anterior optic tract that connects the LO and ME to the AOTU (AOT; Fig. 7b–b″; diameter = 25.2 ± 3.1 µm).

#### Antennal lobes

**Fig. 8:**
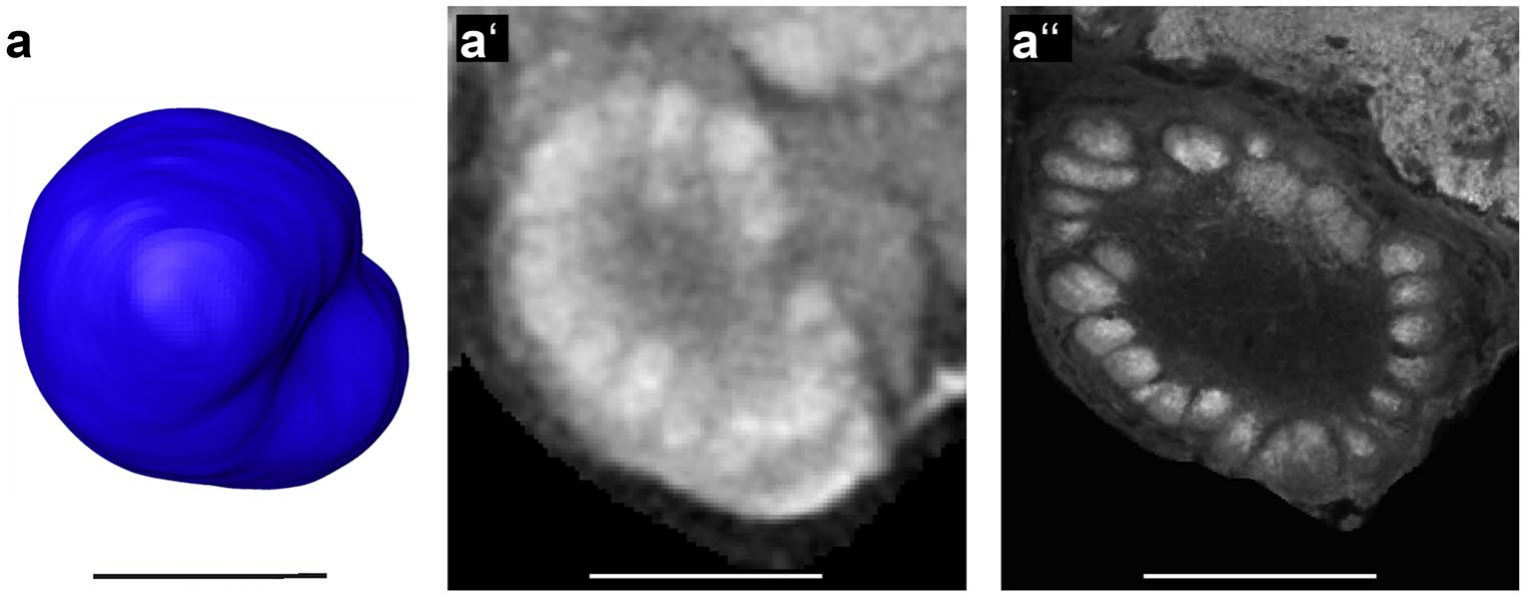
Antennal Lobes. (**a**) Anterior view of shape-based average of surface reconstruction showing the left antennal lobe (AL). (**a′**) Anterior view of one slice of the micro-CT scans showing the AL. (**a″**) Frontal confocal slice of a synapsin stained *B. terrestris* brain showing the AL including single glomeruli. Scale bars = 200 µm

The antennal lobes (AL; Fig. 8) appear as hemispherical protrusions from the ventral brain surface on each side of the esophageal foramen. Olfactory glomeruli were clearly visible in both micro-CT and anti-synapsin stained preparations (Fig. 8a′, a″).

### Volumetric analysis

**Fig. 9:**
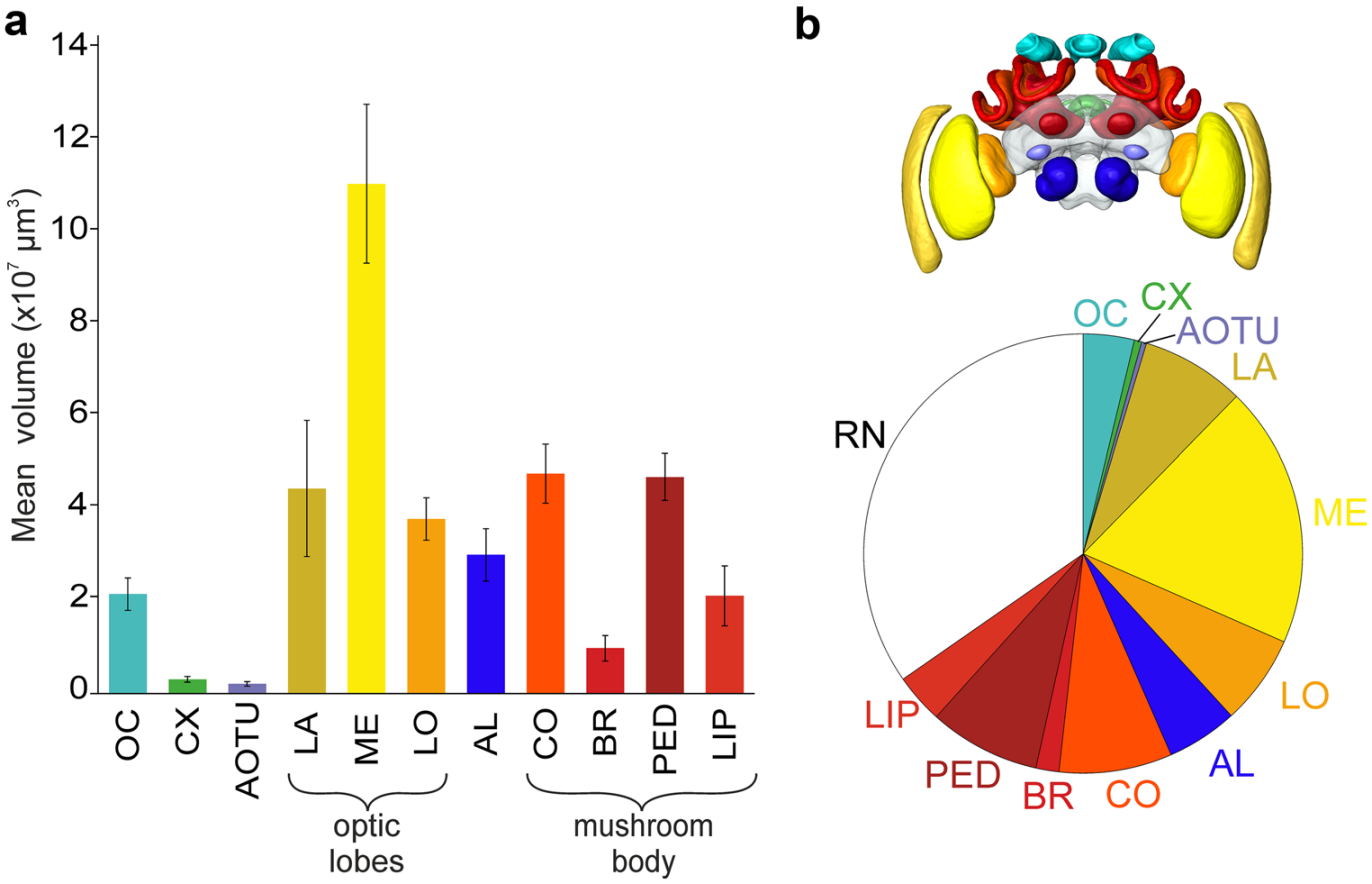
Volumetric analysis of the bumblebee brain. (**a**) Mean volume and standard deviation of the different neuropils in the ten individual bumblebee brains. For better visibility of the smaller volumes the remaining neuropils were left out here. (**b**) Upper illustration shows the shape-based average of reconstructed neuropils. The lower figure highlights the proportion of the reconstructed neuropils. Neuropils: antennal lobes (AL), anterior optic tubercle (AOTU), basal ring (BR), central complex (CX), collar (CO), lamina (LA), lip (LIP), lobula (LO), medulla (ME), ocellar synaptic plexi (OC), peduncle (PED), and remaining neuropils (RN)

The volumetric analysis (Table 1; Fig. 9a) is based on the segmentation of our data set of ten brains and is reported as mean volume ± standard deviation. For structures that appear in both hemispheres, the volumes were measured separately for each side. In addition, the neuropil volumes were calculated as a percentage of all reconstructed neuropils (mean percentage ± standard deviation; Table 1). To make the data more comparable to published data from other species, the percentage of the volume of each reconstructed neuropil was also calculated excluding the lamina (LA) and ocellar synaptic plexi (OC), as these structures are missing in most other standard brains.

**Table 1:** Volume of neuropils of the *Bombus terrestris* standard brain and the ten individual brains. Mean neuropil volumes and standard deviation of 30 segmented brain compartments in ten brains (mean volume, SD (µm^*3*^)). Additionally, the volumes/standard deviations of the standard brain neuropils (standard brain (µm^*3*^)), and the relative volumes/standard deviations of the neuropils in the ten individual brains, either including all neuropils (relative volume (%), SD (%)), or excluding lamina and ocellar plexi (Relative volume without LA, OC, SD (%)) were calculated. Last column: relative volume of all neuropils in standard brain. Neuropils: left and right is signified by -l and -r respectively, antennal lobe (AL), anterior optic tubercle (AOTu), medial basal ring (MBR), lateral basal ring (LBR), lower division of the central body (CBL), upper division of the central body (CBU), central complex (CX), medial collar (MCO), lateral collar (LCO), lamina (LA), lateral lip (LLIP), medial lip (MLIP), lobula (LO), mushroom body (MB), medulla (ME), paired noduli (NO), ocellar synaptic plexi (OC), optic lobes (OL), protocerebral bridge (PB), pedunculus and lobes (PED_LOB), remaining neuropils (RN)

Neuropil	Mean volume (µm^3^)	SD (µm^3^)	Standard brain (µm^3^)	Relative volume (%)	SD (%)	Relative volume without LA, OC (%)	SD (%)	Standard brain (%)
RN	1.99 × 10^8^	2.16 × 10^7^	2.31 × 10^8^	34.7	1.8	39.1	1.8	34.5
OC	2.16 × 10^7^	3.54 × 10^6^	2.40 × 10^7^	3.8	0.5	/	/	3.6
AOTu-r	1.01 × 10^6^	2.82 × 10^5^	1.93 × 10^6^	0.2	0.04	0.2	0.04	0.3
AOTu-l	1.10 × 10^6^	2.26 × 10^5^	1.92 × 10^6^	0.2	0.02	0.2	0.03	0.3
AL-r	1.52 × 10^7^	2.93 × 10^6^	1.67 × 10^7^	2.6	0.3	3.0	0.3	2.5
AL-l	1.50 × 10^7^	2.79 × 10^6^	1.71 × 10^7^	2.6	0.3	2.9	0.3	2.6
**CX**	*3.09 × 10*^*6*^	*6.45 × 10*^*5*^	*3.91 × 10*^*6*^	*0.5*	*0.1*	*0.6*	*0.1*	*0.6*
PB	4.11 × 10^5^	1.33 × 10^5^	3.50 × 10^5^	0.1	0.02	0.1	0.03	0.1
CBU	2.04 × 10^6^	3.66 × 10^5^	2.71 × 10^6^	0.4	0.1	0.4	0.1	0.4
CBL	4.66 × 10^5^	1.03 × 10^5^	6.61 × 10^5^	0.1	0.02	0.1	0.02	0.1
NO	1.71 × 10^5^	4.29 × 10^4^	1.89 × 10^5^	0.03	0.01	0.03	0.01	0.03
**OL**	*1.93 × 10*^*8*^	*3.67 × 10*^*7*^	*2.30 × 10*^*8*^	*33.5*	*3.9*	*29.2*	*2.6*	*34.4*
LA-r	2.13 × 10^7^	5.10 × 10^6^	2.48 × 10^7^	3.7	0.4	/	/	3.7
LA-l	2.33 × 10^7^	9.67 × 10^6^	2.44 × 10^7^	4.0	1.3	/	/	3.7
ME-l	5.56 × 10^7^	9.28 × 10^6^	6.86 × 10^7^	9.6	0.8	10.9	1.0	10.3
ME-r	5.53 × 10^7^	8.02 × 10^6^	6.75 × 10^7^	9.6	0.8	10.8	0.9	10.1
LO-r	1.91 × 10^7^	1.96 × 10^6^	2.21 × 10^7^	3.3	0.2	3.8	0.3	3.3
LO-l	1.88 × 10^7^	2.65 × 10^6^	2.27 × 10^7^	3.3	0.4	3.7	0.5	3.4
**MB**	*1.26 × 10*^*8*^	*2.07 × 10*^*7*^	*1.42 × 10*^*8*^	*21.9*	*2.6*	*24.7*	*2.8*	*21.2*
LCO-l	1.34 × 10^7^	1.51 × 10^6^	1.56 × 10^7^	2.3	0.1	2.6	0.2	2.3
LCO-r	1.30 × 10^7^	1.84 × 10^6^	1.61 × 10^7^	2.3	0.3	2.6	0.3	2.4
MCO-r	1.06 × 10^7^	1.33 × 10^6^	1.25 × 10^7^	1.9	0.1	2.1	0.2	1.9
MCO-l	1.08 × 10^7^	1.70 × 10^6^	1.28 × 10^7^	1.9	0.2	2.1	0.2	1.9
LBR-r	2.59 × 10^6^	6.71 × 10^5^	2.88 × 10^6^	0.4	0.1	0.5	0.1	0.4
LBR-l	2.65 × 10^6^	9.07 × 10^5^	2.96 × 10^6^	0.4	0.1	0.5	0.1	0.4
MBR-r	2.20 × 10^6^	4.80 × 10^5^	2.76 × 10^6^	0.4	0.1	0.4	0.1	0.4
MBR-l	2.42 × 10^6^	7.05 × 10^5^	2.47 × 10^6^	0.4	0.1	0.5	0.1	0.4
PED_LOB-l	2.34 × 10^7^	2.74 × 10^6^	2.62 × 10^7^	4.1	0.3	4.6	0.3	3.9
PED_LOB-r	2.37 × 10^7^	2.35 × 10^6^	2.57 × 10^7^	4.1	0.3	4.7	0.3	3.8
LLIP-r	5.40 × 10^6^	1.15 × 10^6^	4.70 × 10^6^	0.9	0.1	1.1	0.1	0.7
LLIP-l	4.73 × 10^6^	1.88 × 10^6^	5.78 × 10^6^	0.8	0.2	0.9	0.3	0.9
MLIP-r	5.93 × 10^6^	1.91 × 10^6^	5.36 × 10^6^	1.0	0.3	1.1	0.3	0.8
MLIP-l	5.21 × 10^6^	1.57 × 10^6^	5.89 × 10^6^	0.9	0.2	1.0	0.2	0.9

The *B. terrestris* standard brain shows that the OLs are the largest part of the reconstructed areas, occupying 33.5% (29.2% without LA and OC). Within the OL, the ME takes-up the largest volume, followed by the LA and, as the smallest of the three neuropils, the LO. The second biggest neuropil in the bumblebee brain are the MBs taking up 21.9% (24.7% without LA and OC) of the total brain volume. The PED and the corresponding lobes (VL, ML) are the largest neuropils of the MB, followed by the CO, the LIP and, as the smallest part, the BR. The next smaller neuropils are the ALs, which take up 5.2% (5.9% without LA and OC) of the total brain area and is in the range of the OC (3.8%). The smallest neuropils are the AOTUs with 0.4% (0.4% without LA and OC) and the entire CX with 0.5% (0.6% without LA and OC). The RN involve a large percentage of the brain with 34.7 % including all neuropils and 39.1% excluding LA and OC.

### Registration of neuronal data into the standard brain

Standard brains offer the possibility to serve as a reference frame for neuronal data. The current standard procedure to stain individual neurons is injection of a tracer that can be either fluorescent itself or can be labeled with a fluorescent dye. Specimens are then scanned using a confocal microscope to obtain an image data stack.

**Fig. 10:**
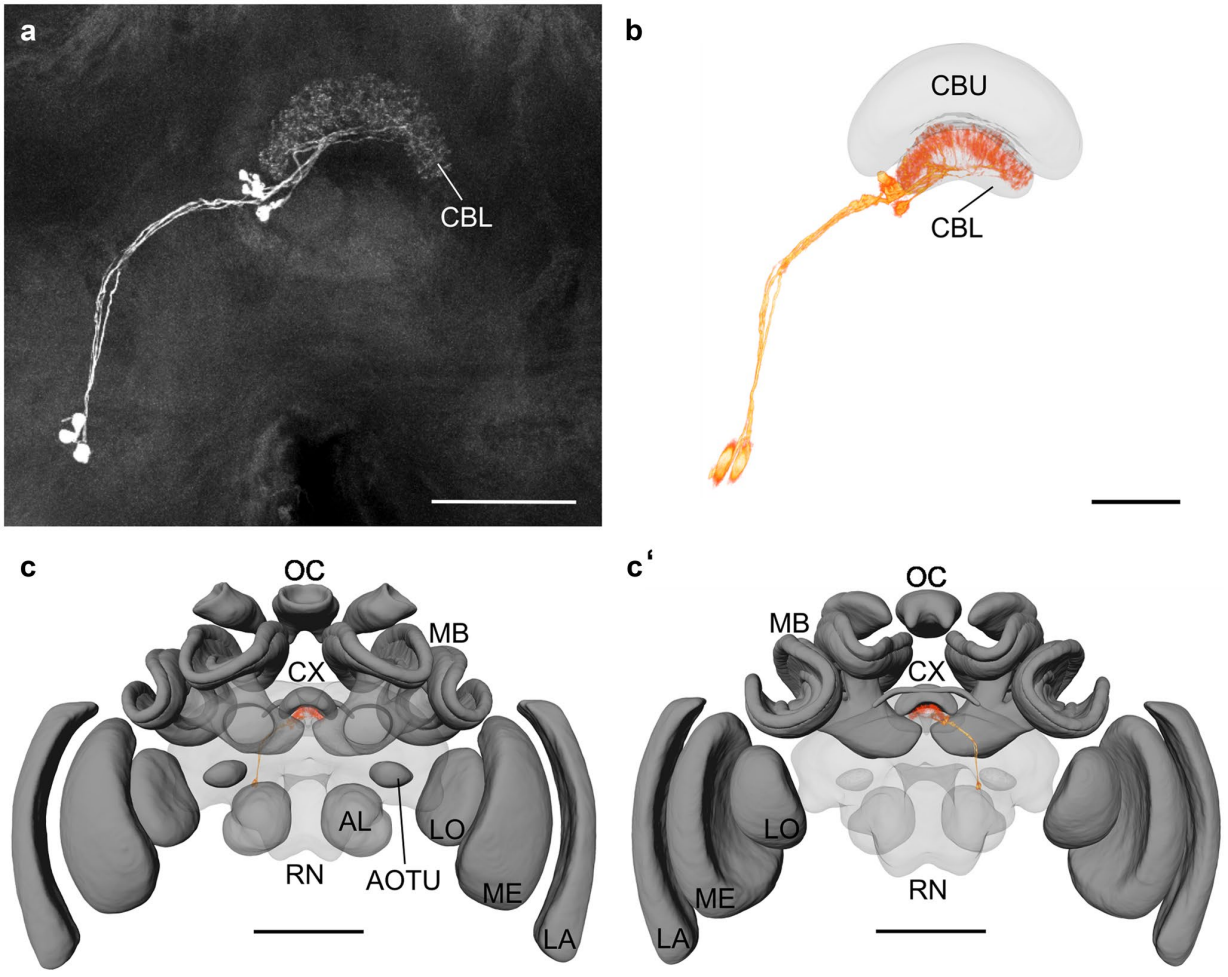
Registration of neurons into the standard brain. (**a**) Projection view of a confocal scan of neurobiotin/Streptavidin-Alexa568 labeled tangential neurons in the central complex. (**b**) Direct volume rendering of tangential neurons registered into the standard brain. To show the individual branches of the neurons in more detail, only the CBU and CBL of the standard brain have been illustrated here. (**c**) Anterior and (**c′**) posterior view of the registered tangential neurons in the entire standard brain to show the localization of the neurons. Neuropils: antennal lobes (AL), anterior optic tubercle (AOTU), lower division of the central body (CBL), upper division of the central body (CBU), central complex (CX), lamina (LA), lobula (LO), mushroom body (MB), medulla (ME), ocellar synaptic plexi (OC), remaining neuropils (RN). Scale bars = 100 µm (**a** and **b**), 1000 µm (**c** and **c′**)

We used a specimen containing neurobiotin/Streptavidin-Alexa 568 labeled tangential neurons of the lower division of the central body (CBL) (Fig. 10a). We reconstructed three neuropils in close spatial relationship to the neurons: CBL, peduncle, and antennal lobe. These neuropil reconstructions were then registered onto the standard brain using affine and elastic registrations. The resulting registration parameters were then applied to the confocal scan data (for details see methods; Fig. 10b, c, c′).

## Discussion

Here we present not only the first 3D standard brain atlas of bumblebees but also the first 3D insect brain atlas based on micro-computed tomography (micro_CT) data. Our data set provides a platform for future neuroanatomical research into the bumblebee brain and demonstrates the usability and advantages of micro-CT for 3D brain standardization.

### Comparison to other hymenopteran standard brains

Standard 3D brains of hymenopteran insects have been previously created for the honeybee *Apis mellifera* (Brandt et al. 2005) and the jewel wasp *Nasonia vitripennis* (Groothuis et al. 2019) based on immunostainings against neuropil markers and confocal laser scanning microscopy. To compare the resolution of the three hymenopteran standard brains, we measured the absolute extent of the 3D-Models in voxels (which differ from the numbers reported in the respective publications that refer to the size of the bounding boxes, exceeding the actual data). To make our data set more comparable to the other two, we removed the ocellar synaptic plexi and the laminae from the model of the bumblebee brain for these measurements (Table 2).

**Table 2:** Resolution of Hymenopteran standard brain models. All dimensions are given in the order x, y, z

Species	Number of voxels	Voxel size (µm)	Total size (µm)	Publication
*Apis mellifera*	471 × 269 × 83	3.9 × 3.9 × 8.1	1837 × 1049 × 672	Brandt et al. (2005)
*Nasonia vitripennis*	1362 × 796 × 134	0.45 × 0.45 × 1.9	613 × 358 × 255	Groothuis et al. (2019)
*Bombus terrestris* (without OC, LA)	602 × 353 × 191	3.9 × 3.9 × 3.9	2348 × 1378 × 745	This publication
*Bombus terrestris* (including OC, LA)	704 × 452 × 191	3.9 × 3.9 × 3.9	2746 × 1763 × 745	This publication

In the x- and y-dimensions, the number of voxels of the *Bombus* micro-CT standard brain ranges between the *Apis* and the *Nasonia* standard brain. It has 128% (x) and 131% (y) of the voxels of the *Apis* standard brain and 44% (x and y) of the voxels of the *Nasonia* standard brain. In the z-dimension, the *Bombus* standard brain has more voxels compared to either the *Apis* (230%) or the *Nasonia* (142%) standard brain. The *Bombus* standard brain in its current version is therefore in a resolution range that allows for registration of individual 3D reconstructed neurons, as has been done in the honeybee before (e.g., Rybak et al. 2010). The numbers of individually reconstructed neuropils in the three hymenopteran standard brains are 21 in *Nasonia*, 22 in *Apis*, and 30 in this study in *Bombus*. These numbers reflect differences in the neuroanatomy of the species, resolution, completeness of the raw data, and the focus of the researchers. While in the *Nasonia* study, there was no clear division of the calyx into lip, collar, and basal ring, the high-resolution permitted individual reconstruction of the sub-compartments of the AOTU (Groothuis et al. 2019). In addition, the lateral horns were included in the *Nasonia* atlas. In the honeybee standard brain, the two subdivisions of the central body were reconstructed as one neuropil, but the PB and the AOTU were not included. Our *Bombus* standard brain is the only one of the three that includes the laminae and the ocellar synaptic plexi, therefore offering the possibility for registration of lamina and ocellar neurons into it. The AOTU is included, but its subdivisions were not reconstructed separately.

### Advantages and limitations of the micro-CT technique compared to confocal microscopy

The micro-CT technique has several salient advantages over antibody staining combined with confocal microscopy. A major drawback of the latter technique is that the brain has to be dissected from the head capsule in order to achieve the best possible antibody penetration of the tissue. The dissection can easily lead to tears in the tissue, which then render the sample unusable for the further standardization process. This is particularly problematic with the LA, which in most species is very difficult to separate from the retina, as the majority of the photoreceptors terminate in the LA. Consequently, the LA has only been reconstructed for the entire data sets of two of nine insect standard brains published to date (hawkmoth: el Jundi et al. 2009; cockroach: Wei et al. 2010). In a third species, the Bogong moth, the LA was included but based on only three of the ten specimens in the atlas (Adden et al. 2020). By using micro-CT, there is no necessity to dissect the brain for staining and therefore remains intact. Furthermore, the brain remains in its natural spatial arrangement (stereo geometry) with all parts of the brain, specifically the optic lobes, being preserved. This is especially important in species with large optic lobes and relatively thin and/or long optic stalks, like locusts (Kurylas et al. 2008), crickets (Honegger and Schürmann 1975), and dung beetles (Immonen et al. 2017). In addition to the optic lobes, the proper shape and correct position in space also play an important role for the ocellar synaptic plexi and the antennal lobes, in some species. Both neuropils are only attached to the rest of the brain via thin connections and can therefore easily be pulled off or stretched during preparation and staining procedure. It should, however, be noted that while the tissue remains in a more natural shape, it might still be prone to shrinkage induced by fixation or contrasting treatments.

Another major advantage of micro-CT is the isotropic voxel size. While confocal systems can achieve very high resolutions in x- and y-directions, their resolution in the z-direction is limited by the point-spread function of the system. The micro-CT technique achieves isotropic resolution and therefore provides identical native resolution in the three cardinal planes. Higher resolution sagittal views using confocal microscopy are not possible without sectioning the brain if its lateral extent exceeds twice the working distance of the lens.

The limitations of the micro-CT technique lie currently in the contrast and noise of the generated images. These parameters, in conjunction with the resolution, determine the level of detail that can be extracted from the raw data. Compared to the general tissue staining using phosphotungstic acid (PTA) used to produce the CT scans we used in this study (Smith et al. 2016), the immunostaining against synaptic markers achieves higher levels of contrast, allowing to extract more features, like specific layering of neuropils. Compared to immunostainings, the use of micro-CT for insect brain tissue is a rather new technique, which was first introduced by Ribi et al. (2008). It is therefore likely that in the future, improved contrasting protocols will become available. Indeed, it is important to note that the micro-CT scanner used to produce the raw images used for this publication was limited to a resolution of around 3 μm. However, technological advances and lower prices means there is now increased accessibility to explore insect neuroanatomy using CT scanners reaching a resolution of 0.5 μm or lower. Furthermore, with micro-CT being under constant development it is therefore to be expected that technical contrasting techniques, noise level and resolution will improve.

### Future directions

The *Bombus* micro-CT standard brain can serve as a platform for future neuroanatomical work in the bumblebee, and here, we provide the first example that this technique is useful for creating standardized brain models. Considering the strengths and limitations of both micro-CT and confocal microscopy, it is desirable to combine both techniques in the future. We demonstrate this concept by registration of a confocal image stack of dye-filled neurons into the micro-CT standard brain atlas. A future improvement of this atlas could be to create a synaptic marker-based standard brain and then registering it into the micro-CT based one, to obtain a standard that is high in detail and contrast as well as in shape-fidelity. With increasing spatial resolution of the micro-CT technique in the future, it might become more practical to directly combine neuropil labeling with staining of individual neurons or groups of neurons in the same micro-CT scan. This might lead to a revival of pre-fluorescence tracing and staining techniques, such as Osmium staining, diaminobenzidine (DAB) staining or cobalt chloride tracing, as described by Lehmann and Melzer (2018). It is also conceivable to visualize the recording site of extracellular copper wire electrodes using micro-CT if copper ions are deposited in the tissue as described by Okada et al. (1999). An additional advantage of such labeling techniques, is that specimens can be further processed for transmission light-microscopy and electron microscopy and are therefore well suited for correlative imaging approaches, as illustrated in Handschuh et al. (2013). Assuming that nickel-intensified DAB stainings are also suitable for micro-CT imaging, such a correlative approach could also be extended to fluorescence microscopy, as has been previously done for TEM/fluorescence microscopy (Sun et al. 1998; Held et al. 2020). Taken together, there are multiple ways in which the micro-CT bumblebee standard brain atlas can be used as a common reference for neuroscience research and we anticipate more techniques to be contributed in the future.

## Supplementary information

Below is the link to the electronic supplementary material.

Supplementary file1 (DOCX 1703 KB)

## Data Availability

The Bombus terrestris micro-CT standard brain is available as 3D-model and averaged micro-CT data on insectbraindb.org, species handle https://hdl.handle.net/20.500.12158/SIN-0000010.3

## Declarations

### Research involving human and animal participants

All applicable international, national, and/or institutional guidelines for the care and use of animals were followed.
