# Supplementary material for: A micro-CT-based standard brain atlas of the bumblebee
Source: Cell Tissue Res. 2021 Jun 28;386(1):29–45. doi: 10.1007/s00441-021-03482-z (PMC8526489; doi:10.1007/s00441-021-03482-z)
Supplement: Supplementary file 1 — Supplementary file1 (DOCX 1703 KB) [file 441_2021_3482_MOESM1_ESM.docx]

Supplementary Information


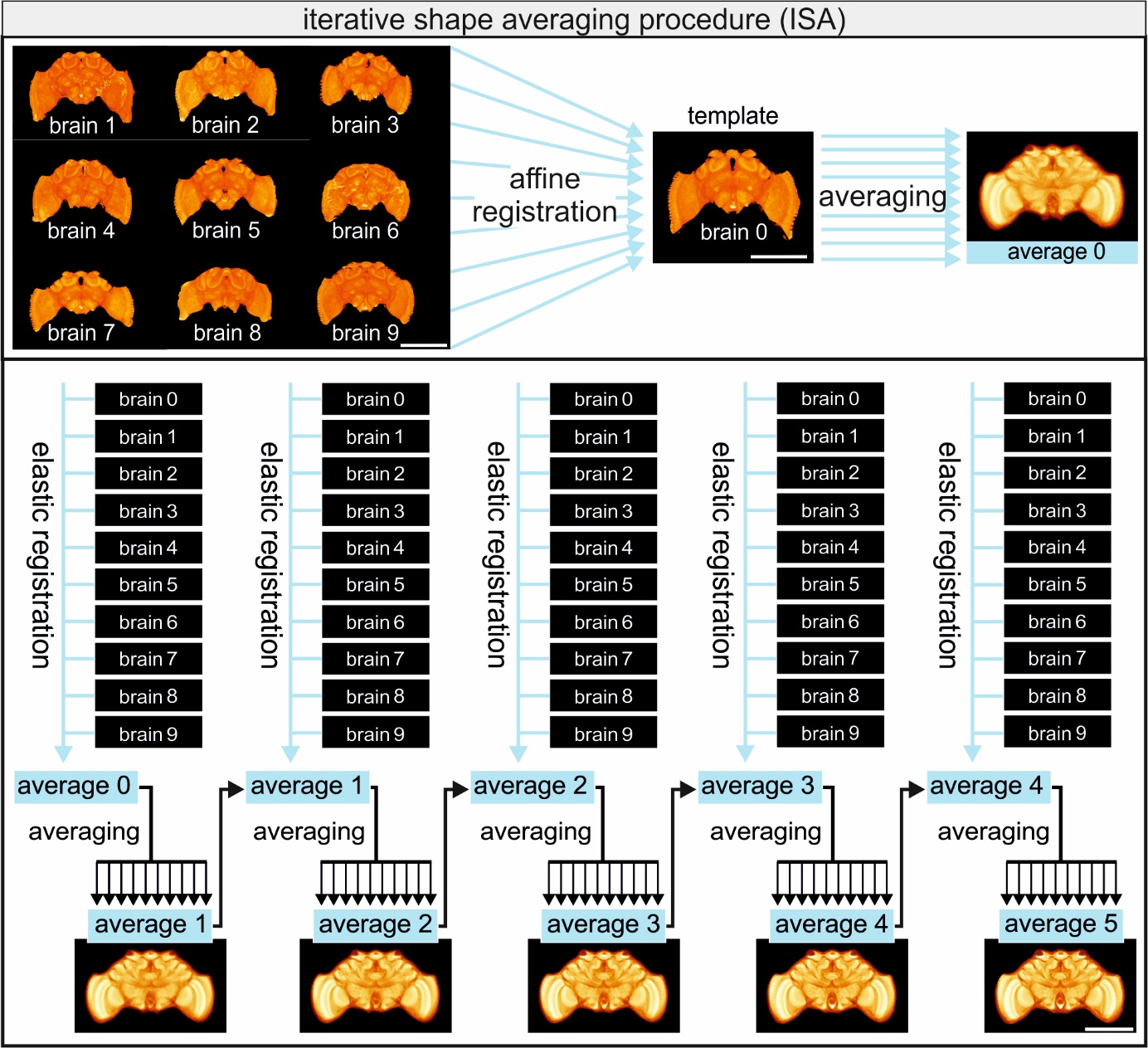


**Fig. S1** Iterative Shape Averaging Procedure. The ISA protocol is a multi-step procedure, in which an affine registration is followed by repeated elastic registrations. First, the brains were aligned to a template brain by affine registration. This procedure compensated for differences in size, position, and rotation between the individual brains. After affine registration, all ten data sets were averaged, and the resulting average brain then served as a template for the subsequent elastic registration. The elastic registration applies local transformations to a 3D grid that is applied to each brain, to optimize the similarity between the images. After the elastic registration of all ten brains, a new average was calculated, which served as a new template for the next iteration. This process was repeated five times, with finer grids in each round. Scale bar = 500 µm


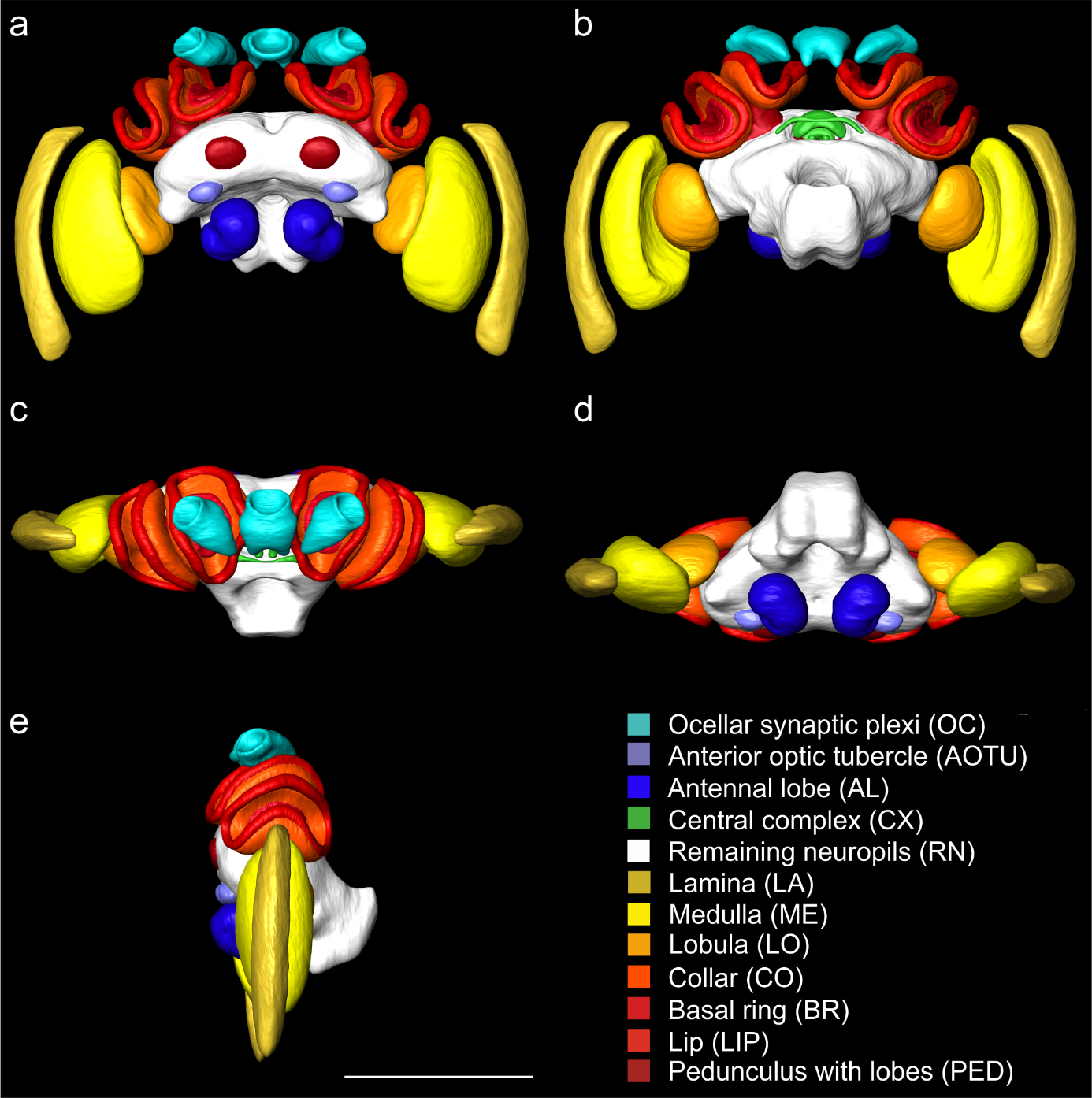


**Fig. S2** Standard brain atlas of Bombus terrestris including the remaining neuropils. Shape based average of surface reconstruction from a frontal (a), posterior (b), dorsal (c), ventral (d), and lateral (e) perspective. The color code at the bottom right represents the colors of the reconstructed neuropils. Scale bar = 1000 µm

**Table S1:** Experimental animals**.** Specimen, age and size information of the experimental animals. Total neuropil volume gives the sum of the volumes of all 30 reconstructed neuropils.

| **Specimen** | **Age of adult bee since pupal emerge** | **intertegula distance (mm)** | **Total neuropil volume (x10^8^ µm^3^)** |
| --- | --- | --- | --- |
| 16Y | 3 | 4.95 | 7.48 |
| 34Y | 12 | 4.93 | 5.74 |
| 115Y | 12 | 3.69 | 6.07 |
| 155Y | 12 | 4.29 | 5.95 |
| 174Y | 3 | 4.55 | 5.51 |
| 222Y | 3 | 3.89 | 4.77 |
| 224Y | 12 | 3.43 | 5.34 |
| 233Y | 3 | 3.74 | 5.46 |
| 240Y | 12 | 4.40 | 5.99 |
| 350Y | 3 | 4.62 | 5.27 |


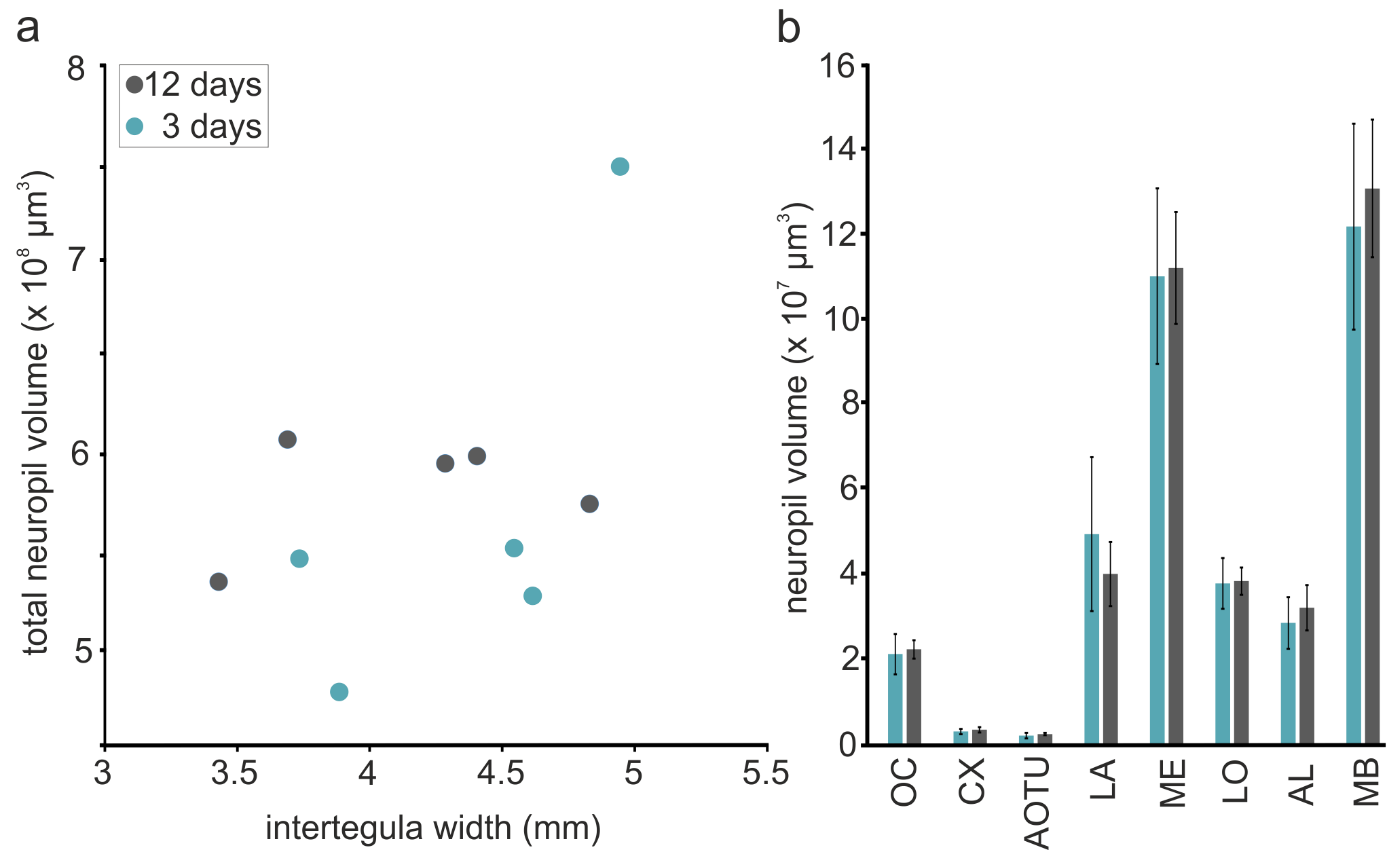


**Fig. S3** Schematic illustration of the evaluation of the experimental animals’ body and brain size. a) Relationship between intertegula width and the total volume of reconstructed neuropils for the individual specimen that were either 3 days (blue dots) or 12 days (grey dots) old since pupal emergence. b) Volumes of reconstructed neuropils shown for the two age groups. Blue bars show the mean neuropil volume (± SD) of the 3-day-old animals, the grey bars show the mean neuropil volume (± SD) of the 12-day-old animals. Neuropils: antennal lobes (AL), anterior optic tubercle (AOTU), central complex (CX), lamina (LA), lobula (LO), medulla (ME), mushroom bodies (MB), ocellar synaptic plexi ocelli (OC). No statistically significant difference between the age groups was found in any of the neuropils (see Table S2)

**Table S2** Statistical evaluation of the experimental animals. Mann-Whitney U-test between the two age groups (3 and 12 days old, Figure S3 b) for intertegula width and the neuropil volumes (antennal lobes (AL), anterior optic tubercle (AOTU), central complex (CX), lamina (LA), lobula (LO), medulla (ME), mushroom bodies (MB), ocellar synaptic plexi ocelli (OC), and remaining neuropils (RN))

| **variables** | **p-value** |
| --- | --- |
| Intertegula width | 0.421 |
| OC | 0.548 |
| LA | 0.548 |
| ME | 0.691 |
| LO | 0.691 |
| AOTU | 0.222 |
| AL | 0.222 |
| CX | 0.222 |
| MB | 0.222 |
| RN | 0.691 |
